# Metabolic health tracking using Ultrahuman M1 continuous glucose monitoring platform in non- and pre-diabetic Indians: a multi-armed observational study

**DOI:** 10.1038/s41598-024-56933-2

**Published:** 2024-03-18

**Authors:** Monik Chaudhry, Mohit Kumar, Vatsal Singhal, Bhuvan Srinivasan

**Affiliations:** Ultrahuman Healthcare Private Limited, No. 799, V K Paradise Sector2, HSR Layout Bengaluru, Bangalore, Karnataka 560102 India

**Keywords:** Metabolism, Continuous glucose monitoring, Wearables, Digital health, Insulin resistance, Glycemic control, Prediabetes, Non-diabetics, Metabolic Score, Inflammation, Endocrine system and metabolic diseases, Lifestyle modification

## Abstract

Continuous glucose monitoring (CGM) device adoption in non- and pre-diabetics for preventive healthcare has uncovered a paucity of benchmarking data on glycemic control and insulin resistance for the high-risk Indian/South Asian demographic. Furthermore, the correlational efficacy between digital applications-derived health scores and glycemic indices lacks clear supportive evidence. In this study, we acquired glycemic variability (GV) using the Ultrahuman (UH) M1 CGM, and activity metrics via the Fitbit wearable for Indians/South Asians with normal glucose control (non-diabetics) and those with pre-diabetes (N = 53 non-diabetics, 52 pre-diabetics) for 14 days. We examined whether CGM metrics could differentiate between the two groups, assessed the relationship of the UH metabolic score (MetSc) with clinical biomarkers of dysglycemia (OGTT, HbA1c) and insulin resistance (HOMA-IR); and tested which GV metrics maximally correlated with inflammation (Hs-CRP), stress (cortisol), sleep, step count and heart rate. We found significant inter-group differences for mean glucose levels, restricted time in range (70–110 mg/dL), and GV-by-SD, all of which improved across days. Inflammation was strongly linked with specific GV metrics in pre-diabetics, while sleep and activity correlated modestly in non-diabetics. Finally, MetSc displayed strong inverse relationships with insulin resistance and dysglycemia markers. These findings present initial guidance GV data of non- and pre-diabetic Indians and indicate that digitally-derived metabolic scores can positively influence glucose management.

## Introduction

Type 2 diabetes mellitus (T2DM) and metabolic syndrome have an estimated prevalence of 422 million^1^. Similarly, the incidence of prediabetes (intermediate hyperglycemia) is increasing at an aggressive rate with a projected estimate of 8.0% (454 million) by 2030 and 8.6% (548 million) by 2045^1,2^. An elevated circulating glucose state is known to upregulate chronic inflammatory markers, and cause cellular stress such as increased production of reactive oxygen species (ROS) which leads to a condition termed insulin resistance, wherein cells become insensitive to insulin and have lower activity-dependent glucose uptake^3,4^. These physiological and cellular changes propel the individual towards the diabetic “state”, and it is estimated that approximately 5–10% of individuals with pre-diabetes (pre-diabetics for brevity) convert to diabetics per year worldwide^5^. The rate of conversion has a large variation depending on diagnostic criteria and geography.

Interestingly, a considerable proportion of pre-diabetics can revert to normo-glycemia if proper corrective measures are implemented including consistent tracking of blood glucose levels, glycemic variability (GV), and complementing lifestyle modification^6^. The widely accepted American Diabetes Association (ADA) guidelines, therefore, strongly emphasize the adoption of these non-pharmacological management and lifestyle modification techniques as soon as a person is diagnosed as a pre-diabetic^7^. The extension of these health management measures has reached the wellness sector in recent times, attesting to their real-world effectiveness.

Several risk evaluation diagnostics and biochemical tests are used for impaired glucose surveillance including random blood glucose, fasting blood glucose [FBG], 75 g-oral glucose tolerance test [OGTT], and glycated haemoglobin [HbA1c]^8,9^. In recent times, the practice of continuous glucose monitoring (CGM) using subcutaneous sensors has revolutionized the concept of real-time glucose tracking and offered dependable solutions for at-home, free-moving screening^10,11^. CGM-mediated real-time tracking improves the detection of glycemic deviations in basal, night-time, and postprandial conditions, and helps derive correlations between diet and exercise, which forms the cornerstone of lifestyle-driven preventive healthcare. These advantages have accelerated the adoption of CGMs among high-performance athletes, fitness-oriented healthy individuals, and those with impaired glucose tolerance^12,13^. The challenge remains in developing easy-to-understand metrics and user interfaces that promote better adoption and compliance, and enhance the predictive component of interpreted glycemic trends by evidence-based correlation.

The Ultrahuman (UH) M1 platform consists of a CGM sensor, application (app)-based analytics, and timely, on-demand, fitness advice provided by certified experts^14^. The captured glucose data is used to generate the daily user-specific metabolic score (MetSc), which is a holistic snapshot of a user’s daily glucose regulation patterns (see “Methods”)^15^. The app also prompts lifestyle adjustment by providing actionable nudges and alerts to the user. Other CGM-based wellness programs are available from Levels Health, January AI, Nutrisense, to name a few^12^.

In South Asia, especially India, CGMs are predominantly used for diabetes management^16^. While wellness and lifestyle monitoring apps are becoming popular, the precise number of healthy-to-mildly at-risk individuals using CGMs/or user-logged fitness app is currently lacking. Moreover, the data that is usually captured on such platforms include user-uploaded, non-biomarker information such as food logs, step counts, sleep duration, etc., which are not immediately co-relatable to clinical glycemic biomarkers. This gap in biomarker-correlated data is especially crucial for India^17^. A recent national survey, reported the overall prevalence of diabetes to be 11.4%, and for prediabetes at 15.3%, the incidence of which had tripled since the last survey in 2017^18,19^. Furthermore the CUREs longitudinal study, reported that 58.9% of pre-diabetic Indians convert to diabetes over a 10-year period^20^. Additionally, the burden of non-communicable diseases (NCD) was calculated to be 40.7% for hypertension, 39.6% for obesity, 51.6% for abdominal obesity, and 82% for dyslipidemia in urban Indians^18^. Taken together, there is substantial evidence of Indian and South Asians having a high propensity to develop metabolic syndrome and surveillance of this demographic is of critical importance. Finally, there is a lack of population-scale, curated digital data for Indian and South Asian profiles to differentiate between the healthy and at-risk individuals, impacting the design of point-of-care, customized interventions, which is the main premise of non-pharmacological management for diabetes prevention as prescribed by ADA.

Therefore, to address this gap, we undertook a multi-arm, observational study to simultaneously derive GV variables in non-diabetics (healthy) and pre-diabetics; correlate these with established markers of inflammation, stress, and lifestyle indicators of sleep, step count, and heart rate; examine the relationship between these biomarkers and MetSc; and finally obtain profiles of glucose tolerance following a 14-day use of the UH-M1 platform.

## Results

### Participant disposition

**Figure 1:**
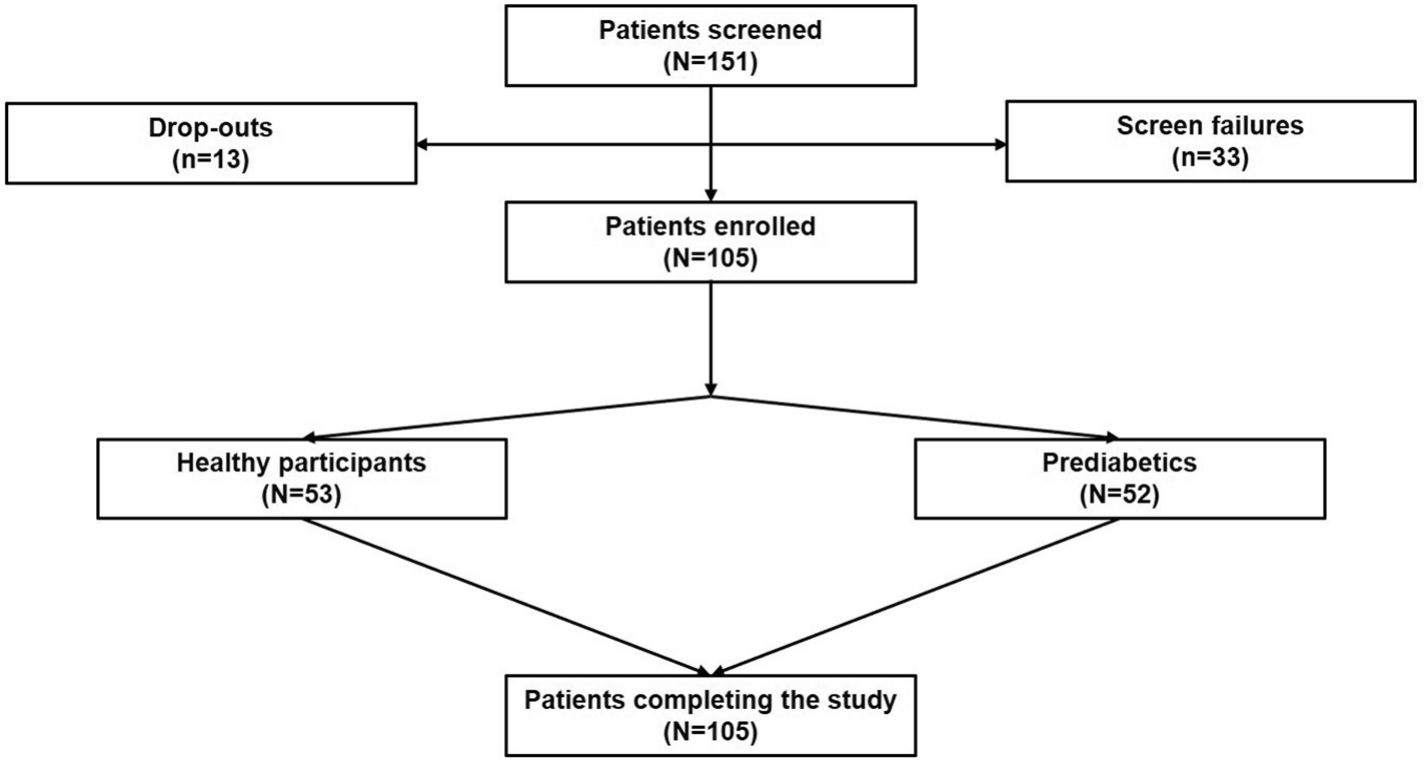
Participant disposition.

A total of 151 individuals were screened of which 105 met the inclusion criteria and participated in the study (Fig. 1). All the 105 enrolled participants completed the study with no drop-outs. Both the individuals with normal ranges of glucose regulation (referred to as healthy group (N = 53)) and individuals presenting with intermediate glucose control (referred to as pre-diabetic group (N = 52)) were well-matched with respect to baseline demographics and clinical characteristics (Supplementary Table S1). The mean BMI was slightly higher in the healthy group, but there was no significant difference between the groups. Physical examination and medical history were well within the inclusion parameters. Although there was a higher prevalence of familial diabetes in the pre-diabetic cohort and a higher rate of familial hypertension in the non-diabetic group, the differences were not statistically supported (data not shown). No serious adverse events were reported during the study.

### Primary outcomes: changes in mean blood glucose and TIRs, across non-diabetic and pre-diabetic groups over time

Over the years, a wealth of studies has contributed to the generation of CGM-derived glycemic datasets^21^. Normal reference ranges of some of these markers are available in small studies with diverse ethnic representation; however, South Asians in controlled studies are usually underrepresented^22^.

**Figure 2:**
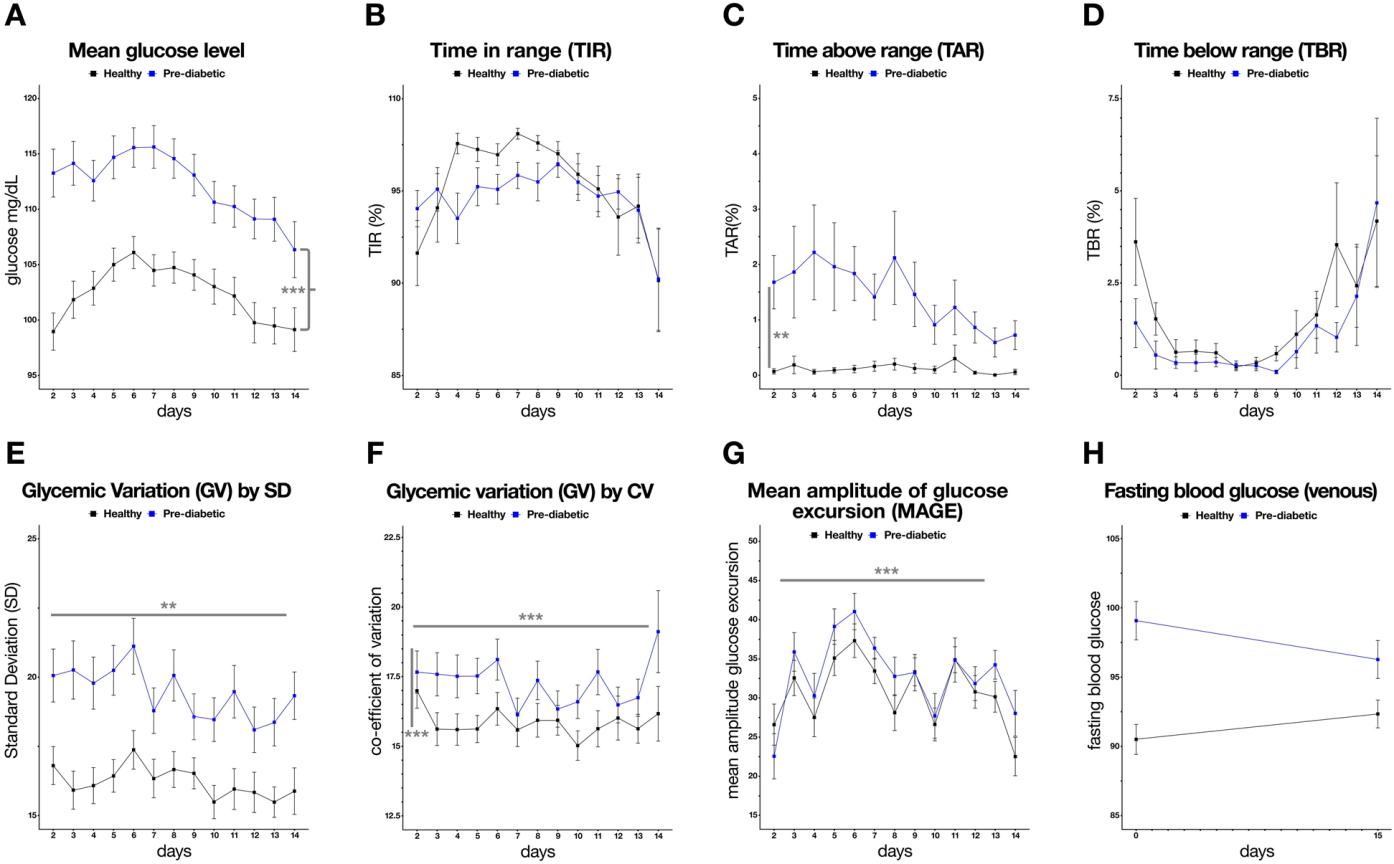
Primary outcome measures in healthy vs. pre-diabetic within the study period. Graphical representation of designated primary outcomes of this study relating to (**A**) mean glucose level, (**B**) time in range, (**C**) time above range, (**D**) time below range, (**E**) glycemic variability calculated by standard deviation, (**F**) glycemic variability calculated by coefficient of variation (CV), (**G**) mean amplitude glucose excursion and (**H**) averaged before and after measure of venous fasting blood glucose. CV: Coefficient of variation; MAGE: Mean amplitude of glycemic excursion; error bars denote: standard deviation. *, **, *** denotes p < 0.05, 0.01 and 0.001, by Two-way ANOVA (see text for details).

In our cohort, we observed a significant difference in the daily mean glucose levels detected by UH-M1 between the healthy (Mean ± SD: 102.4 ± 11.78 mg/dL) and pre-diabetic (Mean ± SD: 112.2 ± 14.25 mg/dL) individuals and this difference extended over the entire duration of 14 days (Two-way ANOVA, main effect, cohort: p < 0.0001; interaction cohort × day: p < 0.01; Fig. 2A). It is noteworthy that there was a significant downward trend over time in mean glucose levels in both groups (main effect, day: p < 0.0001). The mean percentage of CGM-based time in range (TIR per ADA guidelines) between day 2 and day 14 was better in the healthy group (95.3% ± 10.43) than in the pre-diabetic group (94.6% ± 9.4), however, this was not statistically significant (Two-way ANOVA, main effect, cohort: p = 0.91; main effect, day: p = 0.91, Fig. 2B). Furthermore, the TIR values tapered over the period of 14 days, especially towards the end of the study in both the healthy and pre-diabetic cohorts (Fig. 2B). The UH-M1 application uses a relatively tighter target range of 70–110 mg/dL glucose (as compared to the guidance TIR of 70–180 mg/dL). Post-facto calculation for this restricted TIR (rTIR), revealed extremely significant differences between the groups and across days (Two-way ANOVA, main effect, cohort: p < 0.0001; main effect, day: p < 0.0001, interaction cohort × day: p < 0.00001, Supplementary Fig. S2). Pre-diabetic group consistently had lower dwell times in the rTIR range as compared to non-diabetics, and both groups appeared to reach comparable rTIR values by the end of the study period. For time above range (TAR; glucose > 180 mg/dL), there was a statistically significant difference (Two- way ANOVA, main effect, cohort: p < 0.01; main effect, day, not significant (ns), interaction cohort × day: ns) between pre-diabetic (mean 1.4% ± 4.15) and healthy cohorts (mean 0.1% ± 0.72); and the individuals with normal glucose control had negligible hyperglycemic events (Fig. 2C). Interestingly, a clear downward trend was detected in the pre-diabetic group over time (Fig. 2C). There were no distinct trends in time below range (TBR) identifying hypoglycemic events (< 70 mg/dL glucose), in healthy (mean 1.6% ± 6.43) and pre-diabetic (mean 1.0% ± 5.99) group with a higher level of such events in both groups towards the end of the observation period (Fig. 2D).

### Primary outcomes: changes in glycemic variability indices, across non-diabetic and pre-diabetic groups over time

Next, we measured a variety of CGM-derived indices of cumulative glycemic variability in an effort to identify which of the metrics efficiently differentiated between individuals with normal glucose control and those with pre-diabetes. GV, as measured by standard deviation (GV by SD), captured significant across-group differences in the analyses period (19.4 ± 6.51 pre-diabetics vs 16.2 ± 4.85 non-diabetics, Two-way ANOVA, main effect, cohort: p < 0.001; main effect, day: p < 0.001, interaction cohort × day: ns, Fig. 2E). In comparison, GV as measured by the coefficient of variation (GV by CV), had more overlaps between groups, with milder but significantly different values (17.3 ± 5.67 pre-diabetics vs 15.8 ± 4.57 non-diabetics, Two-way ANOVA, main effect, cohort: p < 0.05; main effect, day: p < 0.05, interaction cohort × day: ns; Fig. 2F). Both GV by SD and GV by CV indices displayed a gradual and statistically significant decrease over the trial period in both groups.

Mean amplitude of glycemic excursions (MAGE) values showed across-day improvements, but the level of decrease was not as significant as GV, and there was no distinction between healthy (30.7 ± 15.99) and pre-diabetic (32.9 ± 18.19) groups (Two-way ANOVA, main effect, cohort: p = 0.083; main effect, day: p < 0.0001, interaction cohort × day: ns; Fig. 2G).

Finally, fasting blood glucose levels (FBG, derived by venous blood draw) were monitored at the beginning and end of the study to provide an external anchor point for CGM-derived values. FBG increased modestly from 90.5 ± 7.90 mg/dl at baseline to 92.3 ± 7.28 mg/dl at day 15 for the healthy group, while it decreased from 99.1 ± 10.11 mg/dl at baseline to 96.3 ± 9.84 mg/dl in the pre-diabetics cohort (Fig. 2H). Given the convergent trend of FBG values, there were no significant differences between groups or across time in each group (ANCOVA model with treatment as fixed effect and FBS values at baseline visit as covariate, p = 0.81).

### Secondary outcomes: correlation of CGM-derived glycemic metrics with biomarkers associated with metabolic syndrome

A wealth of evidence supports a strong relationship between stress-related markers such as inflammation, disturbed sleep, reduced physical exercise, and elevated cortisol with the development of intermediate, yet high-risk conditions like prediabetes^23–25^. Several data points are also available for the Indian/South Asian demographic group which used traditional glucose measurements^26^. To address this gap, we carried out a correlation analysis for sleep, stress, inflammation, heart rate, and step count in our cohort with the seven established CGM indices: J-index, high blood glucose index (HBGI), low blood glucose index (LBGI), average daily risk range (ADRR), MAGE, mean of daily differences (MODD) and continuous overall net glycemic action (CONGA)^27^. Here we highlight specific results with all data presented in Supplementary Tables S2–S5.

**Figure 3:**
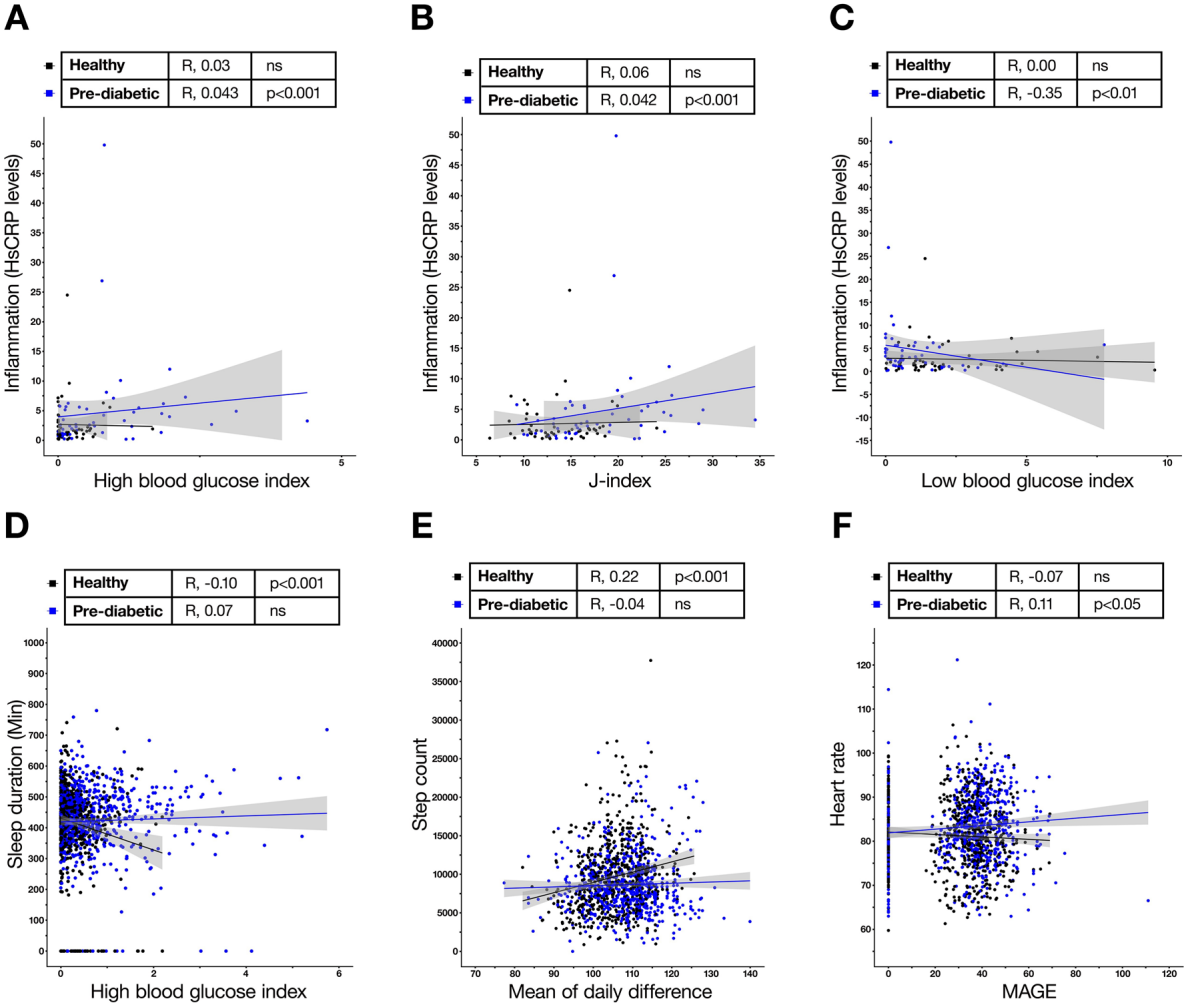
Correlation of CGM-derived glycemic metrics with biomarkers associated with metabolic syndrome. Graphical representation of selected correlation analyses between inflammation, sleep duration, step count and heart rate with glycemic variability indices. R—correlation coefficient. Linked to Supplementary Tables S2–S5.

The strongest correlation was found between GV indices and inflammation as measured by serum Hs-CRP levels (Supplementary Table 2, Fig. 3A–C). The J index, HBGI, and LBGI revealed a strong positive correlation in individuals with pre-diabetes, but not in healthy individuals. Surprisingly, there was little correlation with stress as measured by serum cortisol levels and any of the GV indices in either group (Supplementary Table S3). This may be because GV was calculated over 2–14 days whereas cortisol levels were measured on day 0 and day 15. Sleep duration was negatively correlated with HBGI in non-diabetic but not pre-diabetic group (Supplementary Table S4, Fig. 3D). Interestingly, CONGA, a measure of cumulative glucose fluctuations, was negatively correlated with sleep data in both groups. Fitness tracker-derived motility and sleep metrics showed weak but significant correlations. There was a significant positive correlation between ADRR, MODD, and step count in non-diabetics (Supplementary Table S5, Fig. 3E), while heart rate was positively correlated with ADRR and MAGE in the pre-diabetic group (Supplementary Table S5, Fig. 3F).

### Correlations with CGM-derived GV indices with HOMA-IR, OGTT, and HbA1c

While CGM indices have been utilized to develop algorithms to differentiate between healthy and diabetic individuals, studies evaluating the correlation of CGM-derived GV metrics with clinical gold standards that detect impaired glucose tolerance (IGT) and insulin resistance such as HbA1c, OGTT, and HOMA-IR are scarce^28^. To address this gap, we carried out a post-hoc analysis, correlating the factors recorded in our study (Table 1). For all clinical biomarkers, daily glycemic variability (J-index) revealed strong positive correlation in pre-diabetics. The HBGI count in both healthy and pre-diabetics was a consistent measure of elevated OGTT (measured on day 6) and HbA1c levels. However, HBGI correlated with increased HOMA IR in pre-diabetics cohort only implying that insulin resistance is a feature of IGT space. LBGI showed some interesting correlations with OGTT and HOMA-IR in the healthy group, perhaps indicating that hypoglycemic events requiring glucose mobilization are more tightly regulated in normal glucose tolerance regimes. Specifically, in healthy participants, the glucose fluctuations as captured by the ADRR or MODD are likely better predictors of developing insulin resistance. In summary, there is a difference in the type of glycemic parameters that an individual with healthy glucose control and those with pre-diabetes should focus on when gleaning CGM-derived insights.

**Table 1:** Correlation between Glycaemic variability indices and HbA1C, OGTT, and HOMA-IR (PP Population).

Glycaemic Variability Indices	Statistics	HbA1c %		OGTT		HOMA-IR	
		Healthy (N = 53)	Pre-diabetic (N = 52)	Healthy (N = 53)	Pre-diabetic (N = 52)	Healthy (N = 53)	Pre-diabetic (N = 52)
J index	Correlation coefficient	0.17	0.22	− 0.12	0.39	− 0.15	0.35
	p-value	0.0781	0.0265	0.2377	0.0001	0.3092	0.0169
Low blood glucose index	Correlation coefficient	− 0.16	0.13	0.21	− 0.05	0.47	− 0.06
	p-value	0.1150*	0.2078*	0.0370	0.6403	0.0010	0.6965
High blood glucose index	Correlation coefficient	0.19	0.22	− 0.10	0.39	− 0.08	0.38
	p-value	0.0498*	0.0284*	0.3322	0.0001	0.5791	0.0096
Average daily risk range	Correlation coefficient	0.01	0.32	0.10	0.29	0.45	0.14
	p-value	0.9122*	0.0009*	0.3352	0.0030	0.0017	0.3530
Mean amplitude of glucose excursion	Correlation coefficient	− 0.09	0.09	− 0.01	0.05	0.09	− 0.13
	p-value	0.3382*	0.3671*	0.9313	0.6439	0.5346	0.3857
Mean of daily differences	Correlation coefficient	0.14	− 0.03	− 0.07	0.08	− 0.33	0.04
	p-value	0.1485*	0.7867*	0.5091	0.4094	0.0270	0.8012
Continuous overall net glycaemic action	Correlation coefficient	0.03	0.19	− 0.00	0.02	− 0.01	− 0.03
	p-value	0.7910	0.0496	0.9843	0.8603	0.9346	0.8472

p-value based on Pearson correlation & Spearman correlation test. * represent Spearman correlation test.

### Correlations of MetSc with clinical biomarkers of dysglycemia, insulin resistance and activity

MetSc was developed as an all-encompassing snapshot of glucose tolerance and by extension, of the glycemic fitness of UH-M1 users. While this proprietary score is composed of weighted contributions from an individual’s GV, TIR, and mean glucose values, validation against clinical biomarkers of glucose dysregulation was necessary. We initially monitored the average MetSc across days in both groups. As shown in Supplementary Fig. S3, we found a consistent difference across groups that persisted across the study period, indicating that MetSc can differentiate between individuals with prediabetes and controls. As per study design, participants were not mandatorily expected to act on the app-based nudges (see Supplementary Table S6) and hence, we did not see an appreciable change in group averaged MetSc over the study period. Next, we tested the correlation of MetSc with the clinical biomarkers gathered for both pre- and non-diabetic groups. As shown in Table 2, MetSc had an extremely strong negative correlation with inflammation (HsCRP), HbA1c, OGTT, and HOMA-IR for participants with pre-diabetes. For the non-diabetic group, significant negative correlations were found between OGTT and HOMA-IR only. As a counterpoint, MetSc did not show any correlation with single-snapshot FBG levels in either group, attesting to it’s cumulative informational quality. In the fitness domain, MetSc in both groups was weakly correlated with step count, heart rate, and sleep duration. Heart rate (being tightly regulated), displayed the expected negative correlation with MetSc and was statistically significant for both groups. Interestingly, a small but significant positive correlation was found between sleep duration and MetSc in the healthy group, with a paradoxical weak, negative correlation between sleep and MetSc in the pre-diabetes group.

**Table 2:** Correlation between metabolic score and stress (by cortisol), inflammation (by Hs-CRP), HbA1C%, OGTT values, HOMA IR, fasting glucose levels, and fitness variables (PP Population).

Metabolic Score with Sleep, Stress, Inflammation, HbA1C %, OGTT, HOMA-IR and GMI	Statistics	Healthy (N = 53)	Pre-diabetic (N = 52)
Stress	Correlation coefficient	− 0.05	0.16
	p-value	0.6023*	0.1142*
Inflammation	Correlation coefficient	− 0.16	− 0.38
	p-value	0.2748*	0.0055*
HbA1C (%)	Correlation coefficient	− 0.15	− 0.36
	p-value	0.1397	0.0002
OGTT	Correlation coefficient	− 0.27	− 0.42
	p-value	0.0056*	< 0.0001*
HOMA-IR	Correlation coefficient	− 0.37	− 0.43
	p-value	0.0113*	0.0036*
Fasting blood glucose	Correlation coefficient	0.01	− 0.15
	p-value	0.9186	0.1189
Step count	Correlation coefficient	− 0.004	0.07
	p-value	0.908	0.0978
Heart rate	Correlation coefficient	− 0.13	− 0.08
	p-value	0.0006	0.0380
Sleep duration	Correlation coefficient	0.12	− 0.08
	p-value	0.0014	0.0356

p-value based on Pearson correlation & spearman correlation test. * represents Spearman Correlation test.

## Discussion

The study cohort was representative of an urban, young adult Indian population who were in the overweight-to-obese range which constitutes a third of adults as per the National Family Health Survey (NFHS-5) conducted in 2021^29^. This is also the population most prone to developing pre-diabetes and diabetes in India^18,19^. To our knowledge, this is the first study that provides CGM-derived guidance values of glycemic control in Indians with either healthy glucose regulation and those with pre-diabetes within the 25–50 years age bracket. Furthermore, our extended analyses revealed that of the multiple glycemic indices used in the field, specific GV indices are more relevant for healthy versus the pre-diabetics group in their correlation to clinical benchmarks of glucose control such as HbA1c, OGTT, and insulin resistance by HOMA-IR. Interestingly, MetSc mirrors many of the trends in glycemic dysfunction associated with pre-diabetes and offers a dynamic, easy-to-understand metric that can generate personalized information. Finally, we report that inflammation had the strongest positive correlation with glycemic indices in pre-diabetic group indicating that significant metabolic dysregulation is already underway in these individuals. This supports the notion that glucose tolerance regimes are most likely a contiguous spectrum, rather than discrete states of non-diabetes, pre-diabetes, and diabetes.

Digital health tracking studies like GLITTER, Twin Precision Nutrition (TPN) Program, and ambulatory glucose profiling (AGP) have demonstrated the power of CGM use in making real-time interventional decisions like dietary- or exercise changes, dosage changes of insulin, etc.^30–32^. Within India, electronic health (e-health) and mobile health (m-health) initiatives have been successfully used to provide support, motivation, and directional suggestions to large cohorts to make healthier lifestyle choices^33–35^. Internationally, large cohort studies have been undertaken like the Dehghani Zahedani et al.^35^ (Sugar.AI initiative), that show that a 10-day CGM app-based tracking regime paired with controlled food choices can significantly promote healthier metabolic-oriented choices in healthy and at-risk individuals^35^. The gap lies in reference data of CGM-derived glycemic metrics for healthy Indians (and by extension South Asians), which were either not the focus of clinical studies like GLITTER and Twin, or underrepresented in North American and European study cohorts. With a demographic contribution of over a fifth of the world’s population, and being a high-risk group for developing metabolic syndromes, this group represents an important resource for gathering natural history, baseline evidence and should receive increased surveillance.

It is important to note that we focussed on an urban, and relatively younger demographic for this initial benchmarking study. Older individuals (> 40–50 years) generally get diagnosed with pre-diabetes and related metabolic conditions with higher frequency as a result of increased health check-ups and awareness of NCDs. However, the trend of younger, working South Asians with undetected metabolic syndromes is on the rise^18^. It is well known that lifestyle changes are most efficacious in altering the metabolic health of younger adults, and hence is a key focus area for wearable deployment and of this study.

In terms of primary outcomes of the study, we found a consistent and significant difference in mean glucose levels between the two groups with no significant differences in TIR per the broader ADA guideline range. Interestingly, the rTIR per UH-M1 guidance of a target range of 70–110 mg/dL was significantly improved in both groups and across all days, highlighting that it is possible to derive useable information in a context-specific manner to impact glucose control positively. CGM-based metrics of GV as measured by SD, CV, and MAGE also showed significant improvement over time in both groups. However, the FBG data alludes to the fact that for more lasting changes it is necessary to deploy UH-M1 tracking for a period longer than 14 days and with more concerted translation of app-nudges to lifestyle changes.

Inflammation being the strongest correlative factor with glycemic indices in the pre-diabetic cohort confirmed the fact that there is already significant metabolic dysregulation in this group. This is of particular importance as a clear association between cardiovascular disease and prediabetes has emerged over the past few years^36^. Furthermore, Indians have been known to have a higher HsCRP level both across individuals who are healthy and with pre-diabetes^37,38^. In this study, pre-diabetic participants displayed a trend of having roughly twice the levels of inflammation as compared to healthy participants even though the cohort was comparably overweight. This is in dramatic contrast to the cortisol data, which indicates comparable stress levels in the study cohort.

In the domains of sleep duration, heart rate, and step count, the data seemed to have relevance only in the healthy group. Although not in the remit of this current study, it is possible that multi-modal analyses of data for the pre-diabetic group could identify sub-populations with specific sleep disturbance patterns that have stronger correlation with impaired glucose control. Step count and heart rate were found to have weak correlations with GV in this study. It should be considered that other coexisting factors may also influence these relationships. For healthy individuals, a recent study reported every 1000 step increase per day was correlated with a blunted GV the following day but not within the same day^39^. Per protocol, this study analysed within-day GV with step count and heart rate which may be the reason for the weaker associations seen in this study. Nevertheless, the positive correlation of step count with mean daily differences is a useful indicator of daily swings which can potentially be leveraged to optimally fuel for exercise sessions in healthy individuals.

The aim of analysing several CGM-derived metrics such as J-index, CONGA, MAGE, ADRR and MODD along with HBGI and LBGI was to identify the best metric to use for tracking either cumulative glucose dysregulation or fluctuations in either group. For example, at a cellular level, the strong correlations between inflammation and HBGI and LBGI indicates that high available sugar impact cells directly, likely triggering an intracellular stress response. However, sleep, which is more neurologically dependent, seemed to share modest but significant relationship with only with variables such as CONGA indicating a more cumulative contribution of glycemic regulation to this readout (Fig. 3 and Supplementary Tables S2–S5). The protocol was designed to analyse across days’ differences between groups for all metrics, but given the known relationships between the variables, it may be plausible to infer trends. For instance, given the known linkage between MODD, CONGA, MAGE with GV by SD, it is likely that with a higher MAGE in individuals most likely also predicted higher CONGA etc.^40^. Further investigation is required to satisfactorily explain all trends observed.

An important post-hoc analysis conducted was aimed to address a vital gap in point-of-care surveillance. With busy lifestyles, people often miss conducting wellness check-ups which use gold-standard biomarkers (HbA1c, OGTT, and HOMA-IR) for evaluating glucose tolerance. Hence, by the time systemic symptoms appear, an individual has already progressed to an advanced state within the IGT spectrum. Our results indicate that daily GV (J-index) was a strong proxy in the pre-diabetes group of all three clinical parameters in this population. This relationship did not hold true in the healthy participant group indicating a need to attribute differential weightage to these indices as per specific diagnosis. The HBGI in both healthy and pre-diabetics was a consistent measure of elevated OGTT and HbA1c. However, hyperglycaemic events correlated with increased HOMA IR indicating that insulin resistance was a feature of the IGT space specifically. Instead, in healthy participants, the daily glucose swings as captured by the ADRR or MODD was a better predictor of insulin function, possibly due to optimal glucose utilization during exercise etc. We hope that these relationships will be explored in larger controlled and real-world cohorts to determine the best continuous and personalized proxies for biomarkers of glucose dysfunction.

Wellness/risk score calculators offer an easy-to-understand metric for laypeople to appreciate the risk of developing various conditions. Personalized wearables and digital health monitoring devices are accompanied by aggregate, algorithmic scores to serve as a daily handle for a user to track his/her habits. Only a handful of these scores have been validated using cohorts in trial settings and benchmarked to accepted clinical biomarkers. To our knowledge, our study is novel in its approach to clinically validate MetSc and the results indicate that the score is an effective digital proxy for IGT and insulin resistance in the population studied. Given its relevance to glucose tolerance and insulin resistance, it is plausible to imagine an expanded use case in other ethnic groups.

There were two main limitations of the study, firstly the short duration of CGM use (14-day period) and secondly the interaction of the user with app-interface which can change lifestyle or habits in response to real-time data. Though the study design did not impose mandatory changes in diet, sleep and exercise in response to data collected, it is not possible to rule out that users, who had full access to real-time CGM data, would not have altered their habits and acted on app-nudges. At a group level this alteration in habits did not change the average daily MetSc within the study period (see Supplemental Fig. S3), indicating that 14 days may have been minimally sufficient to glean indications of the positive impact of UH-M1-based tracking. A longer observational study, with more directive mandate of using app-suggestions is required to derive more substantive conclusions on lifestyle changes or the data mining of real-world, non-controlled evidence with larger cohort. Other limitations included under-recruitment of females, capturing basic sleep duration using the Fitbit activity tracker, which did not have refined measurement of sleep epochs which could have uncovered subtle relationships to GV and pre-diabetes. This also holds true for opposing correlation patterns of sleep duration with metabolic score. It is not clear how a high MetSc is linked with poor sleep, in pre-diabetics at the same time that good sleep coincides with high MetSc in non-diabetics. A hardware limitation of the platform involves reliance on one type of CGM sensor^41^.

With this study, we offer an initial foundation for further exploring valuable dynamics of GV with daily activities as well as metabolic parameters in individuals with healthy glucose control and pre-diabetic individuals. The findings underscore the value of using CGMs for wellness and preventive surveillance. Long-term studies will provide more data on these associations and may serve as a guide to personalised management by making adequate lifestyle and if required, pharmacological changes.

## Methods

### Study design and participants

This prospective, two-arm, parallel-group observational study was conducted across multiple urban diabetes clinics and hospitals (N = 9) across the states of Delhi, Karnataka, Telangana, Gujarat, and Tamil Nadu in India (see Supplementary Table S7). The enrolment period ranged from September 2022 to December 2022. The overarching aim of this study was to assess CGM-derived GV indices and their correlation with clinical biomarkers in healthy and pre-diabetic individuals to generate reference data on glycemic health for this age- and geographic-group. The dataset would also be used to (a) investigate MetSc correlation with well-established clinical biomarkers of stress, sleep, inflammation, insulin resistance, and glucose intolerance, and (b) form the basis of updating MetSc. MetSc is a proprietary algorithmic output that was developed by Ultrahuman Pvt Ltd., for the purpose of metabolic fitness tracking and management^15^.

Participants (males and females) were included in the study if they were between 25 and 50 years of age (both inclusive) and had body mass index (BMI) within 20–30 kg/m^2^ range. They were required to comply with the advised use of CGM (Abbott FreeStyle Libre1^42^, activity tracker (Fitbit Inspire 2)), and the UH Application. The exclusion criteria consisted of a history of acute or subacute infection (within the last three months) and chronic illnesses (including T1 (Type 1)- and T2DM, and cardiac disease), anemia, endocrine disorders, and autoimmune conditions. Individuals taking antimicrobials, including antibiotics, antivirals, and antifungals were also ineligible for participation. Participants were not provided any controlled food plan or restricted to maintain a specific daily schedule, food choices, exercise regimes. It was also not mandatory for the participants to interact with the UH-M1 beyond the minimum required to capture glucose values via near-field communication or act on the application-based nudges provided. A sample of the app-nudges and suggestions can be found in Supplementary Table S6.

The study was conducted in compliance with the International Conference of Harmonization/Good Clinical Practice guidelines (ICH-GCP) and the Declaration of Helsinki. The study protocol was reviewed and approved by the ethics committees of all the participating centres. Subjects voluntarily signed a written informed consent form prior to participation and were allowed to withdraw from the study at any time. This study is registered in the Clinical Trials Registry—India (CTRI/2022/08/044808).

### Study procedure

Random sampling method was used to recruit eligible subjects. During the screening visit (between − 3 and − 1 day from the baseline [inclusion] visit), a detailed medical, medication-related, and family history was acquired. Demographic data, anthropometric measurements, and vital signs were recorded and blood samples were obtained to estimate FBG, and glycated HbA1c levels. Potential participants also underwent an OGTT test. Based on the results obtained, the subjects were then screened for eligibility and those selected were divided into two groups: healthy/non-diabetic (FBG: 79–99 mg/dl; HbA1c: 4.0–5.6% and 2-h plasma glucose during 75-g OGTT below 140 mg/dL) and pre-diabetics (FBG: 100–125 mg/dl; HbA1c: 5.7–6.4% and 2-h plasma glucose during 75-g OGTT: 140–199 mg/dL) based on the ADA criteria of Screening and Diagnostic Tests for Prediabetes^7^.

At day 0 (baseline visit), the eligibility was reconfirmed by repeating the OGTT and a general physical examination. Details regarding the CGM and UH-M1 application were also explained to the participants during this visit. The app was installed on the smartphone of the subject, and he/she was trained on the features of the app and its use. Once the subject was familiar with the app, the CGM was attached to the upper arm (preferably left) and activated followed by the initializing of the app. The participants were asked to follow their regular daily routine and log (food information) the same on the UH-M1 app daily. He/she was instructed to contact the investigator or the team in case of any difficulties while using the app.

Adverse reactions (if any) were planned to be coded using the MedDRA central coding dictionary, version 25. All medications were to be coded using the WHO-DD, September 1, 2019, or later. Preferred ATC coding was planned to be applied to encode medications use.

A second follow-up visit was arranged between days 5 and 7 of the trial period. The tests conducted on this day included an OGTT and a general physical examination. This OGTT value was used for correlation analyses reported in data tables. For participants with any concomitant indigestion, gastric irritation, or vomiting, the OGTT visit was postponed to a few days after, however within the trial period. Data collection ended on day 14 of CGM and app use, followed by a final, physical examination and laboratory investigations. In the case of sensor failure (sensor stopped reporting values or widely fluctuating measurements) the endpoint occurred earlier. This session was termed as the “End of study” (EOS) visit. In addition, the participants also completed a satisfaction feedback form and a Pittsburgh Sleep Quality Index (PSQI) sleep self-assessment questionnaire during this visit^43^. Subjects experiencing any temporary health issues, technical difficulties in using CGM, CGM data collection failure, or non-compliance with the app were discontinued/withdrawn from the study.

### Study endpoints

The primary endpoints included CGM-based glucose indices over 14 days period such as the mean glucose levels as described by a 24-h profile during 2 weeks; time in glucose ranges (TIR: 70–180 mg/dL for “acceptable” diabetes glucose range; TAR: time-above-range > 180 mg/dL and TBR: time-below-range < 70 mg); GV as measured by the standard deviation (SD) and the coefficient of variation (CV); and the mean amplitude of glycaemic excursions (MAGE), defined as the arithmetic mean of the amplitude of glucose excursions that are greater than the standard deviation of the glucose values. In addition, for the preventive/wellness use case, the UH-CGM application employs a tighter target range of 70–110 mg/dL; and hence it was also computed for the healthy and pre-diabetic groups post-facto after study completion. Daily MetSc scores were generated for each participant across the study period which were then used for correlation analyses (representative snapshot of MetSc display on the app interface is depicted in Supplementary Fig. S1).

The secondary endpoints included changes in FBG levels from day 0 to 15, the correlation between GV indices and sleep duration, step count, heart rate (acquired from fitness tracker use), and blood-based biomarkers such as stress (serum cortisol), inflammation (serum Hs-C reactive protein (Hs-CRP)). Additional samples to catalogue gut microbiome, and urine metabolites were also acquired for future analyses and are not within the scope of this manuscript.

### Statistical analysis

Data were analyzed between day 2 and 14 of CGM-use to rule out differences in sensor application across participants and known variability of sensor output in the first 24 h of sensor activation^44^. Data were analyzed using the R Software version 4.2.2^45^. Intent-to-Treat (ITT) set (included all subjects who were enrolled in the study) and Per Protocol (PP) set (included all subjects who completed the study procedures as per the planned protocol) were defined for analysing the data. Normality tests were performed to select the appropriate test and the outliers were removed following ± 3SD for normal distributions, and beyond three times lowest and highest interquartile range (IQR) for non-parametric data. Categorical data were presented as frequencies and proportions and compared using the Chi-square test with Yates correction or Fischer's exact t-test, as appropriate. Continuous data were presented as mean with SD or median with interquartile range and were compared using unpaired t-test, or Mann–Whitney U test, as appropriate. In addition to summary statistics, the differences in the primary endpoint results between non-diabetic and pre-diabetic subjects’ groups were compared using statistical models. Least-squares means (LSM), visit differences in LSM, and the corresponding 95% confidence intervals (CIs) for the subject group differences were estimated using the model. Receiver operating characteristic (ROC) curve analysis was used to assess the predictive value of CGM-based GV indices in prediabetes. For the secondary endpoints, Pearson correlation or Spearman coefficients were calculated and presented in graph and tabular outputs to assess the association between the clinical biomarkers, and interstitial glucose. Linear models were also used to explore these associations. All statistical tests were conducted at a 2-sided alpha level of 0.05 and a 2-sided 95% CI was provided.

### Ethical approval

Ethical approval was obtained from each of the clinical trial sites individually. The details of the trial site, ethics committee, and date of approval are provided in a tabular format in Supplementary Table S7.

### Supplementary Information

Supplementary Information 1.

Supplementary Information 2.

## Data Availability

Study outcomes data can be made available upon reasonable request. The M1 and MetSc platform codes and technical details are proprietary assets of Ultrahuman and will not be disclosed. B.S. stands guarantee of the veracity of the study data. Please contact Bhuvan Srinivasan (bhuvan@ultrahuman.com).
