# Supplementary material for: Metabolic health tracking using Ultrahuman M1 continuous glucose monitoring platform in non- and pre-diabetic Indians: a multi-armed observational study
Source: Sci Rep. 2024 Mar 18;14:6490. doi: 10.1038/s41598-024-56933-2 (PMC10948749; doi:10.1038/s41598-024-56933-2)
Supplement: Supplementary file 1 — Supplementary Information 1. [file 41598_2024_56933_MOESM1_ESM.docx]

**Metabolic health tracking using Ultrahuman M1 continuous glucose monitoring platform in non- and pre-diabetic Indians: a multi-armed observational study**

**Authors:** Monik Chaudhry^1,4^ Mohit Kumar^1,2^ Vatsal Singhal^1,2^ and Bhuvan Srinivasan^1,3^

**Supplementary Information**

Supplementary Figures:

Figure S1: Exemplar snapshot of the MS information panel on the M1 platform. Links to Table 2.

Figure S2: Restricted time in range in healthy vs pre-diabetic within the stipulated time-frame. Links to Figure 1.

Figure S3: Average daily metabolic score (MetSc) in participants with normal glucose control vs those with pre-diabetes within the study period. Links to Table 2.

Supplementary Tables:

Supplementary Table S1: Baseline demographics. Links to Figure 1.

Supplementary Table S2: Correlation between Glycaemic variability indices and inflammation (as measured by Hs-CRP). Links to Figure 2.

Supplementary Table S3: Correlation between Glycaemic variability indices and stress (as measured by cortisol). Links to Figure 2.

Supplementary Table S4: Correlation between Glycaemic variability indices and sleep (as measured by FitBit tacker sleep duration). Links to Figure 2.

Supplementary Table S5: Correlation between Glycaemic variability indices and physical activity (as measured by FitBit Step count and Heart rate). Links to Figure 2.

Supplementary Table S6: List of application-based nudges provided to participants

Supplementary Table S7: Table detailing trial sites, ethics committees and dates of approval. Links to methods.

Additional Supplemental Files: 1) STROBE checklist for this observational study. 2) Protocol and CTRI files trial information.

**Supplementary Figures**

**Supplementary Figure S1:**

**
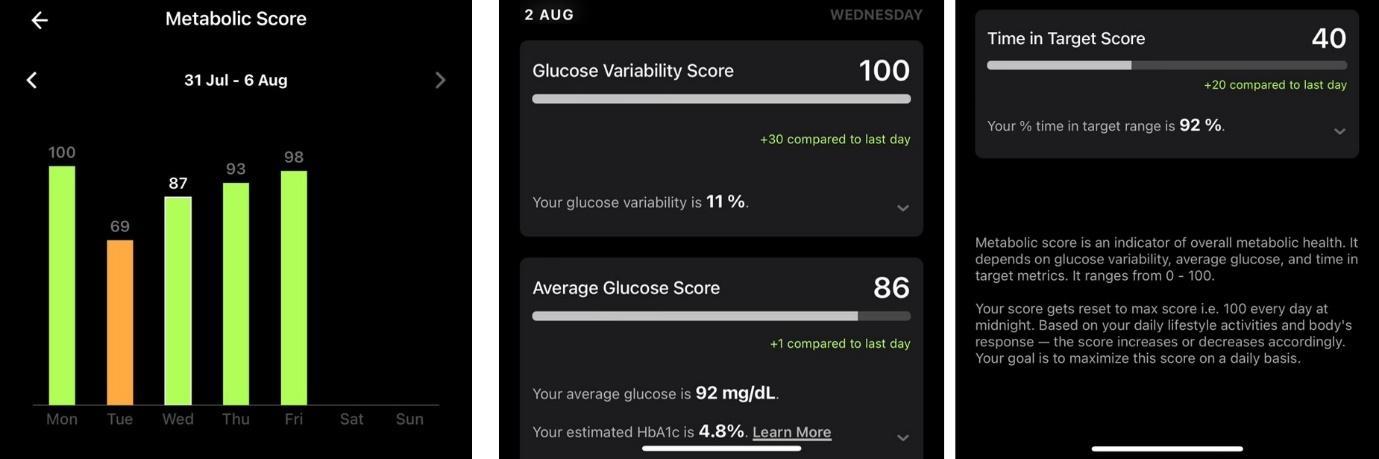
**

**Exemplar snapshot of the MS information panel on the M1 platform.** The user interface provides daily scores of glucose variability, average glucose and time in target range and compares it to the previous day. MS is set to 100 at the start of each day and based on the user’s activity, food intake and lifestyle increases (to reflect good glycemic control) or decreases (to indicate fair or poor glycemic management). User is urged to maximize the score across the day.

**Supplementary Figure S2**

**
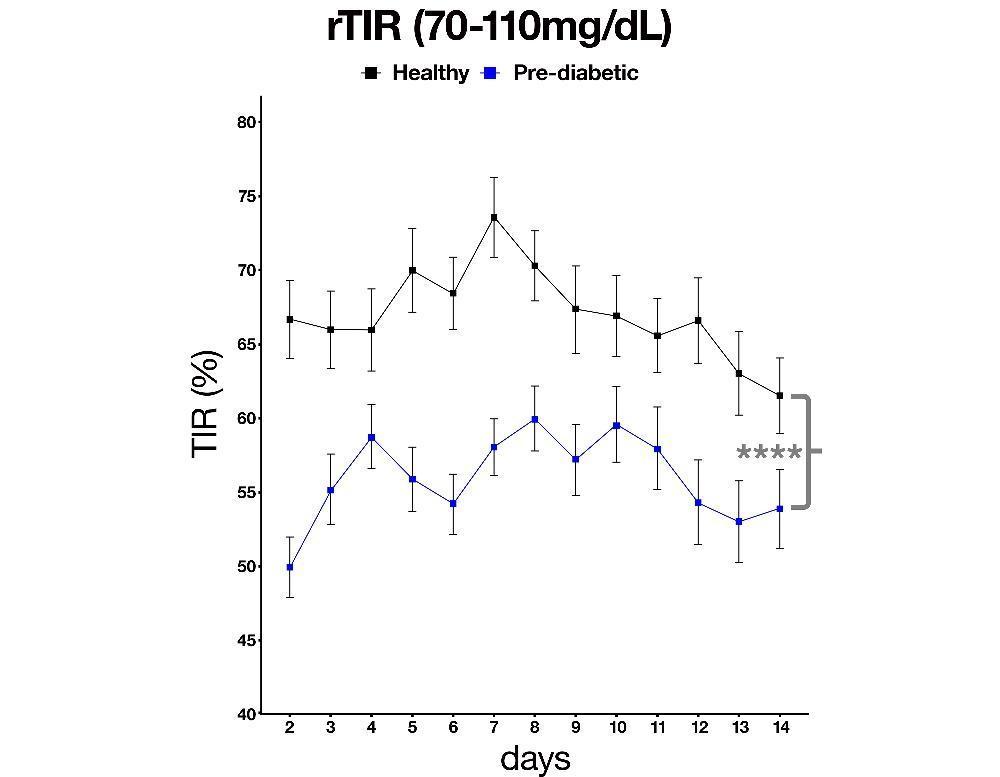
**

**Restricted time in range (rTIR) in healthy vs pre-diabetic within the stipulated time-frame.** UH application adopts a tight time in range as compared ADA guidelines. Grouped data from post-hoc analyses carried out on daily rTIR values for all participants, averaged daily in both groups is shown here to demonstrate the different rTIR in pre- and non-diabetics. Across the study period this rTIR appear to converge for both groups around 55-65%. UH: Ultrahuman, ADA: American Diabetes Association. Statistical analyses: Two-factor ANOVA **(**cohort x day; main effect, cohort: p<0.0001; main effect, day: p<0.0001, interaction cohort x day: p<0. 0.00001). Error bars denote S.D.

**Supplementary Figure S3**
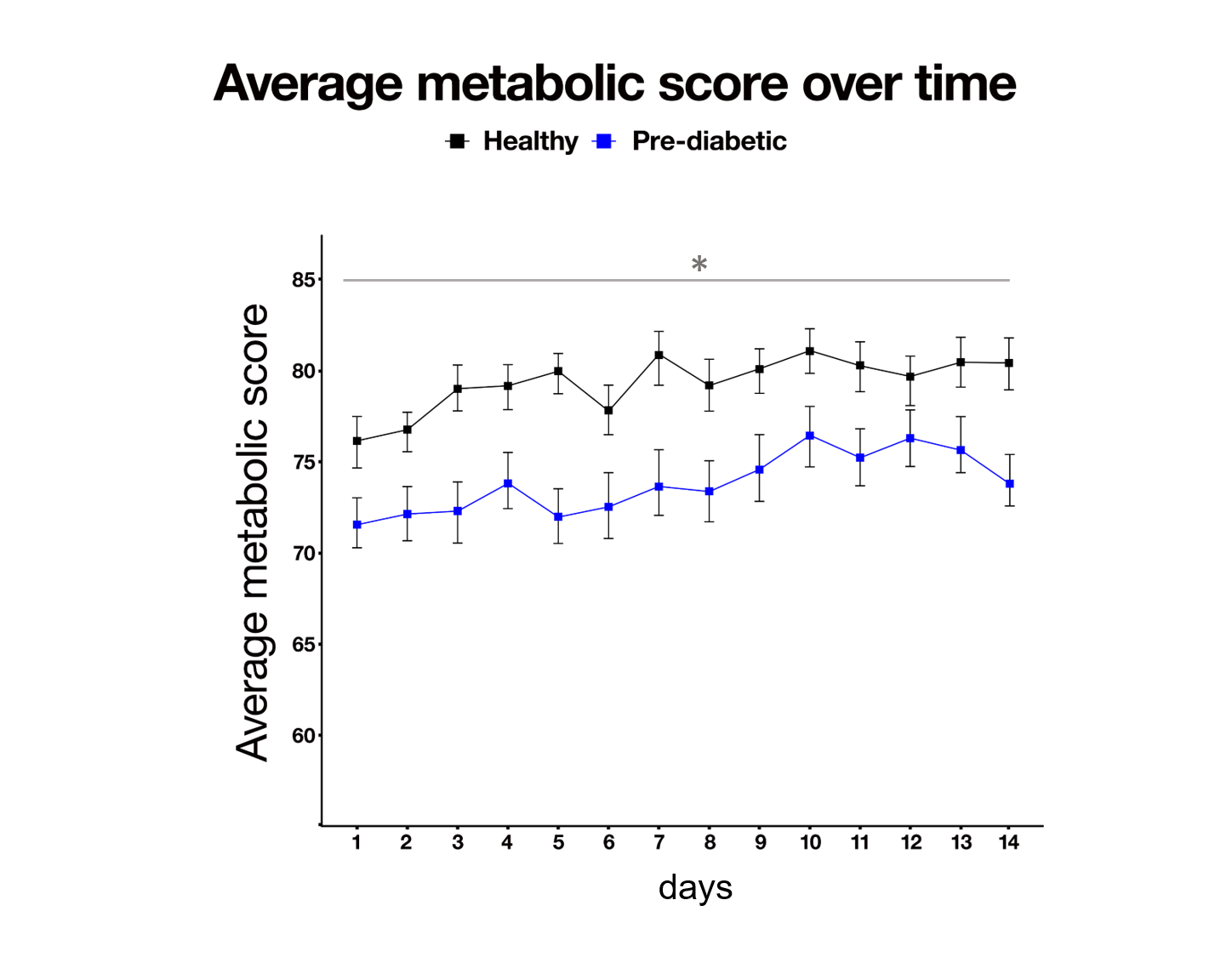


**Average daily metabolic score (MetSc) in participants with normal glucose control vs those with pre-diabetes within the study period.**

Grouped data from post-hoc analyses carried out average metabolic score of participants in both groups, demonstrate a consistent differentiation, however does not appreciable change in 14 days of non-directed, controlled lifestyle habits. Statistical analyses: Two-factor ANOVA **(**cohort x day; main effect, cohort: p<0.05; main effect, day: n.s., interaction cohort x day: n.s). Error bars denote S.E.M.

**Supplementary Table S1: Baseline demographics**

| **Parameter** | **Statistics** | **Healthy   (N=53)** | **Pre-Diabetic   (N=52)** |
| --- | --- | --- | --- |
| Gender n (%) | Male | 39 (73.6) | 45 (86.5) |
| Age (Years) | N* | 53 | 52 |
|  | Mean (SD) | 32.8 (6.56) | 35.6 (6.94) |
|  | Median (Q1, Q3) | 30.0 (28.0, 37.0) | 35.5 (30.5, 41.0) |
|  | Min, Max | 25.0, 47.0 | 25.0, 49.0 |
| BMI (kg/m^2^) | N* | 53 | 52 |
|  | Mean (SD) | 25.571 (2.8634) | 26.456 (2.6311) |
|  | Median (Q1, Q3) | 25.390 (24.050, 27.940) | 26.830 (24.905, 28.410) |
|  | Min, Max | 20.030, 29.760 | 20.260, 29.940 |

BMI: Body mass index; SD: Standard deviation.

**Supplementary Table S2: Correlation between Glycaemic variability indices and inflammation (as measured by Hs-CRP) (PP Population)**

| **Inflammation with   glycaemic variability indices** | **Statistics** | **Healthy  (N = 53)** | **Pre - Diabetic  (N = 52)** |
| --- | --- | --- | --- |
| J index | Correlation coefficient | 0.06 | 0.42 |
|  | p-value | 0.6959 | 0.0020 |
|  |  |  |  |
| Low blood glucose index | Correlation coefficient | -0.002 | -0.35 |
|  | p-value | 0.9876 | 0.0108 |
|  |  |  |  |
| High blood glucose index | Correlation coefficient | 0.03 | 0.43 |
|  | p-value | 0.8162 | 0.0016 |
|  |  |  |  |
| Average daily risk range | Correlation coefficient | -0.05 | -0.12 |
|  | p-value | 0.7448 | 0.3840 |
|  |  |  |  |
| Mean amplitude of glucose excursion | Correlation coefficient | 0.01 | -0.10 |
|  | p-value | 0.9637 | 0.4692 |
|  |  |  |  |
| Mean of daily differences | Correlation coefficient | -0.10 | 0.20 |
|  | p-value | 0.5034 | 0.1492 |
|  |  |  |  |
| Continuous overall net glycaemic action | Correlation coefficient | 0.04 | 0.004 |
|  | p-value | 0.7912 | 0.9772 |
| p-value based on Pearson correlation & spearman correlation test * represent spearman correlation test | | | |

**Supplementary Table S3: Correlation between glycaemic variability indices and stress (as measured by cortisol) (PP Population)**

| **Stress with   glycaemic variability indices** | **Statistics** | **Healthy  (N = 53)** | **Pre - Diabetic  (N = 52)** |
| --- | --- | --- | --- |
| J index | Correlation coefficient | 0.06 | -0.15 |
|  | p-value | 0.5615* | 0.1253* |
|  |  |  |  |
| Low blood glucose index | Correlation coefficient | -0.04 | 0.09 |
|  | p-value | 0.7242* | 0.3663* |
|  |  |  |  |
| High blood glucose index | Correlation coefficient | 0.04 | -0.16 |
|  | p-value | 0.6688* | 0.0970* |
|  |  |  |  |
| Average daily risk range | Correlation coefficient | 0.01 | -0.08 |
|  | p-value | 0.8956* | 0.4105* |
|  |  |  |  |
| Mean amplitude of glucose excursion | Correlation coefficient | -0.09 | 0.06 |
|  | p-value | 0.3760* | 0.5502* |
|  |  |  |  |
| Mean of daily differences | Correlation coefficient | -0.07 | -0.08 |
|  | p-value | 0.4685* | 0.4078* |
|  |  |  |  |
| Continuous overall net glycaemic action | Correlation coefficient | -0.09 | -0.05 |
|  | p-value | 0.3521 | 0.5844 |
| p-value based on Pearson correlation & spearman correlation test * represent spearman correlation test | | | |

**Supplementary Table S4: Correlation between Glycaemic variability indices and sleep duration (PP Population)**

| **Glycaemic variability indices** | **Statistics** | **Healthy  (N = 53)** | **Pre - Diabetic  (N = 52)** |
| --- | --- | --- | --- |
| J index | Correlation coefficient | -0.08 | 0.07 |
|  | p-value | 0.0336 | 0.0872 |
|  |  |  |  |
| Low blood glucose index | Correlation coefficient | 0.01 | -0.05 |
|  | p-value | 0.8938 | 0.2408 |
|  |  |  |  |
| High blood glucose index | Correlation coefficient | -0.10 | 0.07 |
|  | p-value | 0.0082 | 0.0845 |
|  |  |  |  |
| Average daily risk range | Correlation coefficient | -0.07 | 0.06 |
|  | p-value | 0.0768 | 0.1262 |
|  |  |  |  |
| Mean amplitude of glucose excursion | Correlation coefficient | -0.06 | -0.06 |
|  | p-value | 0.1025 | 0.1445 |
|  |  |  |  |
| Mean of daily differences | Correlation coefficient | -0.05 | -0.04 |
|  | p-value | 0.1591 | 0.3318 |
|  |  |  |  |
| Continuous overall net glycaemic action | Correlation coefficient | -0.09 | -0.10 |
|  | p-value | 0.0159 | 0.0115 |
| p-value based on Pearson correlation & spearman correlation test * represent spearman correlation test | | | |

**Supplementary Table S5: Correlation between Glycaemic variability indices and physical activity (step count) (PP Population)**

| **Glycaemic variability indices** | **Statistics** | **Step Count** | | **Heart rate** | |
| --- | --- | --- | --- | --- | --- |
|  |  | **Healthy  (N = 53)** | **Pre - Diabetic  (N = 52)** | **Healthy  (N = 53)** | **Pre - Diabetic  (N = 52)** |
| J index | Correlation coefficient | -0.03 | -0.09 | 0.04 | 0.06 |
|  | p-value | 0.4363 | 0.0174 | 0.3207 | 0.1545 |
|  |  |  |  |  |  |
| Low blood glucose index | Correlation coefficient | 0.04 | 0.01 | 0.11 | -0.06 |
|  | p-value | 0.2966 | 0.7298 | 0.0045 | 0.1100 |
|  |  |  |  |  |  |
| High blood glucose index | Correlation coefficient | 0.004 | -0.10 | 0.05 | 0.05 |
|  | p-value | 0.9173 | 0.0134 | 0.2071 | 0.1768 |
|  |  |  |  |  |  |
| Average daily risk range | Correlation coefficient | 0.10 | 0.004 | 0.15 | 0.07 |
|  | p-value | 0.0082 | 0.9146 | 0.0001 | 0.0847 |
|  |  |  |  |  |  |
| Mean amplitude of glucose excursion | Correlation coefficient | 0.06 | -0.05 | -0.07 | 0.11 |
|  | p-value | 0.0950 | 0.1747 | 0.0520 | 0.0048 |
|  |  |  |  |  |  |
| Mean of daily differences | Correlation coefficient | 0.22 | -0.04 | -0.08 | -0.04 |
|  | p-value | <0.0001 | 0.2973 | 0.0412 | 0.2983 |
|  |  |  |  |  |  |
| Continuous overall net glycaemic action | Correlation coefficient | 0.04 | -0.06 | -0.09 | 0.05 |
|  | p-value | 0.2493 | 0.1449 | 0.0145 | 0.2324 |
| p-value based on Pearson correlation & spearman correlation test * represent spearman correlation test | | | |  |  |

**Supplementary Table S6: Exemplar list of application based nudges provided to UH-M1 platform users**

| **Event** | **Alert/Nudges** |
| --- | --- |
| Glucose level drop below 70mg/dl | Glucose crash detected. Glucose crashes can lead to subsequent hunger peaks. Optimize foods that crash your blood glucose to manage hunger levels. |
| Persistent average glucose notification | Your average glucose for yesterday was xxx mg/dL (+y than the max target for average glucose). This could happen in case you are consuming more carbs or simple sugars than what your body can utilize optimally. |
| Glucose level exceeds 120mg/dl | Your blood glucose is rising. Log food or activity behind this rise. |
| Stable glucose response 1. Diurnal 2. Nocturnal | 1. Nice and stable! You've maintained stable glucose levels so far. This helps improve your energy levels and cognitive performance. 2. Your glucose trends during nocturnal hours (12-6 AM) seem stable and within the healthy range. This helps with optimal sleep performance and recovery. |
| Physical activity prompt | The next 3 hours are primed for physical activity based on your recent fuelling trends. You might see improved workout performance and results for work done during these hours. |
| Reminder to scan | You haven't scanned your sensor in the last 7 hours. Please scan your sensor to avoid missing glucose readings. |
| Missed glucose data (scan detected but no glucose data received) | Your glucose data has not been received in the last 30 minutes. Make sure your glucose sensor is connected to the app. |

**Supplementary S7: Details of trial sites, principal investigators, ethics committee approval dates**

| **Site Name (Name of Ethics Board)** | **Site Address** | **Date of Approval** | **EC Registration Number** |
| --- | --- | --- | --- |
| Aakash Healthcare  Private Limited (Aakash Healthcare Super Speciality Hospital Institutional Ethics Committee) | Aakash Healthcare, Private Limited Hospital Plot Road No. 201, Sector-3, Dwarka, New Delhi -110075 | 11^th^ August 2022 | ECR/1265/Inst/DL/2019 |
| Aadhavvan Diabetes &  Research Centre (Universal Ethics Committee) | No.3, 5th Street, Eswar Nagar, Kodambakkam, Chennai, Tamil Nadu - 600024 | 08^th^ August 2022 | ECR/125/Indt/TN/2013/RR-16 |
| Belgaum Diabetes  Centre (Diabetes Centre Ethics Committee) | Ground and second floor, Maruti street, Belgaum, Karnataka- 590001 | 12^th^ August 2022 | ECR/341/Indt/KA/2021 |
| Suresh Diacare (Bangalore Ethics Committee) | No 723B, 11th Main Rd, 3rd Block, Rajajinagar,  Bengaluru, Karnataka 560010 | 26^th^ September 2022 | ECR/355/Indt/KA/2022 |
| Diacare Research (Shrey Hospital Institutional Ethics Committee) | 1,2 Gandhi Park, near Nehrunagar, Ambawadi,  Ahmedabad, Gujarat- 380015 | 12^th^ August 2022 | ECR/1302/Inst/GJ/2019 |
| Vinaya Hospital, (Ethics Committee Vinaya Hospital) | Vinaya Hospital and Research Centre (a unit  of KIMS), Karangalpady, Mangaluru, Karnataka-575003 | 30^th^ July 2022 | ECR/664/Inst/KA/2014/RR-20 |
| Induss Hospital,  (Induss Hospital Institutional Ethics  Committee) | Induss Hospital, Opp. Kothapet Fruit Market Kothapet, Sri  Sai Shivani Complex, HUDA Complex,  Saroornagar, Hyderabad, Telangana- 500035 | 14^th^ October 2022 | ECR/1606/Inst/TG/2021 |
| Kulkarrnis Medzonne (Bangalore Ethics Committee) | Kulkarrni’s Medzonne, GD Naidu Hall, Mohan Matrix, 450^th^ ,12^th^ cross road, Near Vidya Bharti School, Mahalakshmipuram, Bengaluru, Karnataka. 560086 | 03^rd^ October 2022 | ECR/355/Indt/KA/2022 |
| Naveda Healthcare Centre (Good Society Ethical Research- Institutional Ethics Committee for Biomedical Research) | A-1/81, Sector-8, Rohini, Delhi. 110085 | 02^nd^ August 2022 | ECR/69/Indt/DL/2013/RR-16 |
