# Supplementary material for: Metabolic health tracking using Ultrahuman M1 continuous glucose monitoring platform in non- and pre-diabetic Indians: a multi-armed observational study
Source: Sci Rep. 2024 Mar 18;14:6490. doi: 10.1038/s41598-024-56933-2 (PMC10948749; doi:10.1038/s41598-024-56933-2)
Supplement: Supplementary file 2 — Supplementary Information 2. [file 41598_2024_56933_MOESM2_ESM.docx]

**STROBE STATEMENT: Metabolic health tracking using Ultrahuman M1 continuous glucose monitoring platform in non- and pre-diabetic Indians: a multi-armed observational study**

Monik Chaudhry^1,4^ Mohit Kumar^1,2^ Vatsal Singhal^1,2^ and Bhuvan Srinivasan^1,*^

^All changes in revised version in blue font^

|  | Item No. | Recommendation | Page  No. | Relevant text from manuscript |
| --- | --- | --- | --- | --- |
| **Title and abstract** | 1 | (*a*) Indicate the study’s design with a commonly used term in the title or the abstract | 1,2 | Metabolic health tracking using Ultrahuman M1 continuous glucose monitoring platform in non- and pre-diabetic Indians: a multi-armed observational study |
|  |  | (*b*) Provide in the abstract an informative and balanced summary of what was done and what was found | 2 | Abstract: lines 30-49 |
| Introduction | | | |  |
| Background/rationale | 2 | Explain the scientific background and rationale for the investigation being reported | 5-6 | Line 110-114, 115-120 |
| Objectives | 3 | State specific objectives, including any prespecified hypotheses | 5 | Line 115-120 |
| Methods | | | |  |
| Study design | 4 | Present key elements of study design early in the paper (N/A by Sci Reports format) | 17 | Line 404-434 |
| Setting | 5 | Describe the setting, locations, and relevant dates, including periods of recruitment, exposure, follow-up, and data collection | 17 | Lines 404-415, Supplementary Table S7 |
| Participants | 6 | (*a*) *Cohort study*—Give the eligibility criteria, and the sources and methods of selection of participants. Describe methods of follow-up  *Case-control study*—Give the eligibility criteria, and the sources and methods of case ascertainment and control selection. Give the rationale for the choice of cases and controls  *Cross-sectional study*—Give the eligibility criteria, and the sources and methods of selection of participants | 17-19 | Line 404-472 |
|  |  | (*b*) *Cohort study*—For matched studies, give matching criteria and number of exposed and unexposed  *Case-control study*—For matched studies, give matching criteria and the number of controls per case | NA | NA |
| Variables | 7 | Clearly define all outcomes, exposures, predictors, potential confounders, and effect modifiers. Give diagnostic criteria, if applicable | 20-21 | Line 475-492 |
| Data sources/ measurement | 8* | For each variable of interest, give sources of data and details of methods of assessment (measurement). Describe comparability of assessment methods if there is more than one group | 20-21 | Line 475-492 |
| Bias | 9 | Describe any efforts to address potential sources of bias | NR | Observational study |
| Study size | 10 | Explain how the study size was arrived at | Supplementary file: Protocol | Section 6.1 |

Continued on next page

| Quantitative variables | 11 | Explain how quantitative variables were handled in the analyses. If applicable, describe which groupings were chosen and why | 20-21 | Line 488-509 |
| --- | --- | --- | --- | --- |
| Statistical methods | 12 | (*a*) Describe all statistical methods, including those used to control for confounding | 20-21 | Line 488-509 |
|  |  | (*b*) Describe any methods used to examine subgroups and interactions | 20-21 | Line 488-509 |
|  |  | (*c*) Explain how missing data were addressed | NA, 5 | Line 122-124 |
|  |  | (*d*) *Cohort study*—If applicable, explain how loss to follow-up was addressed  *Case-control study*—If applicable, explain how matching of cases and controls was addressed  *Cross-sectional study*—If applicable, describe analytical methods taking account of sampling strategy | NR | NR |
|  |  | (*e*) Describe any sensitivity analyses | NA | NA |
| Results | | | | |
| Participants | 13* | (a) Report numbers of individuals at each stage of study—eg numbers potentially eligible, examined for eligibility, confirmed eligible, included in the study, completing follow-up, and analysed | 5 | Line 122-124, Figure 1 |
|  |  | (b) Give reasons for non-participation at each stage | 19 | Line 465-467 |
|  |  | (c) Consider use of a flow diagram | - | Figure 1 |
| Descriptive data | 14* | (a) Give characteristics of study participants (eg demographic, clinical, social) and information on exposures and potential confounders | 5 | Line 115-126 and  Supplementary table S1 |
|  |  | (b) Indicate number of participants with missing data for each variable of interest | NA | NA |
|  |  | (c) *Cohort study*—Summarise follow-up time (eg, average and total amount) | NA | NA |
| Outcome data | 15* | *Cohort study*—Report numbers of outcome events or summary measures over time | 5-11 | Line 122-267 |
|  |  | *Case-control study—*Report numbers in each exposure category, or summary measures of exposure |  |  |
|  |  | *Cross-sectional study—*Report numbers of outcome events or summary measures |  |  |
| Main results | 16 | (*a*) Give unadjusted estimates and, if applicable, confounder-adjusted estimates and their precision (eg, 95% confidence interval). Make clear which confounders were adjusted for and why they were included | NA | NA |
|  |  | (*b*) Report category boundaries when continuous variables were categorized | 5-11 | Line 122-267 |
|  |  | (*c*) If relevant, consider translating estimates of relative risk into absolute risk for a meaningful time period | NA | NA |

Continued on next page

| Other analyses | 17 | Report other analyses done—eg analyses of subgroups and interactions, and sensitivity analyses | 7, 10-11 | Lines: 152-160, 243-267 |
| --- | --- | --- | --- | --- |
| Discussion | | | | |
| Key results | 18 | Summarise key results with reference to study objectives | 11 | Line 270-285 |
| Limitations | 19 | Discuss limitations of the study, taking into account sources of potential bias or imprecision. Discuss both direction and magnitude of any potential bias | 16 | Line: 378-390 |
| Interpretation | 20 | Give a cautious overall interpretation of results considering objectives, limitations, multiplicity of analyses, results from similar studies, and other relevant evidence | 16 | Line: 391-396 |
| Generalisability | 21 | Discuss the generalisability (external validity) of the study results | 16 | Line: 391-396 |
| Other information | |  | | |
| Funding | 22 | Give the source of funding and the role of the funders for the present study and, if applicable, for the original study on which the present article is based | 24 | Line: 632 |

*Give information separately for cases and controls in case-control studies and, if applicable, for exposed and unexposed groups in cohort and cross-sectional studies.
